# Cloning, Expression, Isotope Labeling, and Purification of Transmembrane Protein MerF from Mercury Resistant *Enterobacter* sp. AZ-15 for NMR Studies

**DOI:** 10.3389/fmicb.2017.01250

**Published:** 2017-07-07

**Authors:** Aatif Amin, Zakia Latif

**Affiliations:** Department of Microbiology and Molecular Genetics, University of the PunjabLahore, Pakistan

**Keywords:** *Enterobacter* sp. AZ-15, pET31b+, MerF, Size exclusion chromatography, ^1^H-^15^N heteronuclear single quantum coherence, Hg-detoxification, scanning electron microscopy (SEM)

## Abstract

Mercury resistant (Hg^R^) *Enterobacter* sp. AZ-15 was isolated from heavy metal polluted industrial wastewater samples near to districts Kasur and Sheikhupura, Pakistan. 16S rDNA ribotyping and phylogentic analysis showed 98% homology with already reported *Enterobacter* species. The *mer*F gene encoding transmembrane protein-MerF was amplified from genomic DNA and ligated into pET31b+ vector using restriction endonucleases, *Sph*I and *Xho*I. The genetic codons of *mer*F gene encoding cysteine residues were mutated into codons, translating into serine residues by site-directed mutagenesis. Ketosteroid isomerase (KSI), a fusion tag which is present in pET31b+ vector, was used in the expression of *mer*Fm gene. KSI was used to drive the target peptide (MerFm) into inclusion bodies so that the peptide yield and purity were increased. The stable plasmid pET31b+:*mer*Fm was transformed into C43(DE3) *E.coli* cells. The high expression of uniformly ^15^N isotopically labeled-MerFm protein was induced with 1 mM IPTG. The purification of ^15^N-MerFm recombinant protein by Ni-NTA and size exclusion chromatography involved an unfolding/refolding procedure. The two-dimensional HSQC NMR spectra of MerFm protein showed the purity and correct number of resonances for each amide. ^1^H–^15^N HSQC NMR experiment also confirmed that no modification of the tryptophan residue occurred during cyanogen bromide cleavage. A small scale reservoir of Luria Bertani (LB) medium supplemented with 20 μg/ml of HgCl_2_ showed 90% detoxification of Hg by *Enterobacter* sp. AZ-15. The accumulation of Hg on the cell surface of this strain was visualized by scanning electron microscopy (SEM) which confirmed its potential use in Hg-bioremediation.

## Introduction

Mercury toxicity is a worldwide problem to both human and animals. The level of mercury pollution in the environment is being increased day by day due to anthropogenic sources and activities like the discharge of industrial effluent from chlor-alkali industries, mining of metal and incineration of coal (Steenhuisen and Wilson, 2015). It is obvious that both forms of mercury (inorganic and organic) cause cytotoxic and neurotoxic effects to humans and animals (WHO, 2000).

Bacterial detoxification systems have spawned much interest in recent times for their potential usefulness in the bioremediation of environmental contaminants (Silver, 1992). Heavy metals are just one of a variety of contaminants that have appeared in our environment, and quite a few natural resistances to them have already been documented. Plasmid-born resistances to a wide variety of heavy metals have been explored and the genes encoding their resistances have been sequenced (Silver, 1992). The best characterized of these systems is the bacterial mercury detoxification system, the *mer* operon, and in particular those on transposons Tn*21*, Tn*501*, and Tn*5053* (Gilbert and Summers, 1988; Dahlberg and Hermansson, 1995).

Bacteria can be used for bioremediation because they take up mercury *via* membrane potential-dependent sequence-diverged members of the mercuric ion (Mer) superfamily, i.e., a periplasmic Hg-scavenging protein (MerP) and one or more inner membrane-spanning proteins (MerC, MerE, MerF, and MerT), which transport Hg^2+^ into the cytoplasm (Barkay et al., 2003). All the *mer* operons have *mer*T and *mer*P, however, some operons, such as transposon Tn*21*, have *mer*C. The *mer*F is also part of *mer* operons of Gram negative bacteria and is absent in the *mer* operons of Gram positive bacteria. The additional *mer*C gene is located between *mer*P and *mer*A. However, it seems not to be essential for Hg^2+^ resistance since it is absent from Tn*501*, which confers identical Hg^2+^ resistance levels (Summers, 1986).

The enzymatic mercury detoxification system is one of the most remarkable because of its level of sophistication. Mercury is toxic at very low levels, as Hg^2+^ with no known biological benefit. Its toxicity is related to nonspecific reactivity with sulfhydryl groups in proteins and high permeability through biological membranes. Expression of proteins encoding mercury resistance is governed by an operator/promoter region, which interacts with a divergently transcribed MerR biosensor protein. The roles of the gene products designated T (116 amino acids), P (72 amino acids), C (143 amino acids), and A have been worked out using gene deletion studies (Hamlett et al., 1992; Wilson et al., 2000). In detoxification mechanism, the toxic Hg^2+^ atoms are ‘handed off’ from MerP to MerT, which transports Hg^2+^ to the reductase, MerA (Ghosh et al., 1999). More recently, a homolog of MerT, designated MerF (81 residues), has been sequenced from *Alcaligenes* and *Pseudomonas* genera posited to have the same function (Yeo et al., 1998; Sone et al., 2013).

Integral to this transport system are several pairs of cysteine residues, which are known to bind Hg^2+^ in a linear bicoordinate manner. In particular, the motifs -CC-, -CXC-, -CXXC-, and -CXXXXXC- are found in MerT/MerF, MerE. MerP, and MerC, respectively (DeSilva et al., 2002). Mutagenesis of the cysteine residues suggests that only one of the cysteine residues in MerP, Cl7, is important, and only the first -CC- pair in MerT and MerF is important (Powlowski and Sahlman, 1999). In MerT mutation of a cysteine residue in the -CXXXXXC- pair is only slightly detrimental to its transport function, while mutation in the vicinal -CC- residues in either MerT or MerF is detrimental in the transportation (Hobman and Brown, 1997).

MerF is predicted to have two membrane-spanning segments. It has been shown definitively to function as a transporter of mercuric ions into the cell by possession of two vicinal pairs of cysteine residues which are involved in the transport of Hg^2+^ across the membrane and are exposed to the cytoplasm. More importantly, MerF alone is sufficient for the transport of Hg^2+^ across the cell membrane. NMR studies of integral membrane domain and full length MerF from *Escherichia coli* (C41 and C43 cells) have been investigated (Das et al., 2012; Lu et al., 2013; Tian et al., 2014).

Inoculation of contaminated sites with selected or engineered bacteria has often not been satisfactory, partly because the introduced metabolic potential was not the limiting factor for pollutant degradation (Cases and de Lorenzo, 2005). This applies also to mercury resistance, which is ubiquitous in soil and water, even in the Arctic (Barkay and Poulain, 2007; Møller et al., 2011). For the treatment of mercury-contaminated groundwater, pilot experiments have been carried out with the aim to establish bio-barriers in the groundwater where sulfate reducing bacteria (SRB) could adsorb mercury and precipitate it as insoluble cinnabar (Wagner-Döbler et al., 2000; Dash and Das, 2012; He et al., 2015).

In the present study, a major transporter protein of bacterial Hg-detoxification system, MerF isolated from Hg-resistant *Enterobacter* sp. AZ-15 was first time studied and then the potential of selected bacterial strain AZ-15 in the detoxification of Hg (II) was evaluated. These objectives were achieved by (1) the screening of mercury resistant bacteria from polluted natural environment and their 16S rDNA phylogenetic analysis (2) designing the gene construct and expression of mutated *mer*F (*mer*Fm) gene (3) purification of MerFm protein by Ni-NTA and size exclusion chromatography (4) NMR studies of ^15^N isotopically labeled-MerFm protein (5) the analysis of Hg-detoxification potential at lab scale and SEM of selected bacterial cells.

## Materials

Enzymes were purchased from New England Biolabs (www.neb.com) unless otherwise noted, and the oligonucleotides were synthesized by Integrated DNA Technologies (www.idtdna.com). GeneJET genomic DNA purification kit and MAX efficiency® DH5α™ competent *E. coli* cells were purchased from Thermo Fisher© (www.thermofisher.com). Rapid DNA Dephos & ligation kit was obtained from Life Sciences© (www.lifescience.roche.com). Gel extraction kit, miniprep kit for small-scale plasmid preparations and His-tag nickel affinity resin (Ni-NTA) were purchased from Qiagen© (www.qiagen.com). QuikChange lightning site-directed mutagenesis kit was from Agilent Technologies© (www.genomics.agilent.com). Plasmid DNA of pET-31b(+) and bacterial strain OverExpress™ C43(DE3) were purchased from Lucigen© (www.lucigen.com). ^15^N-ammonium sulfate and d_25_-sodium dodecyl sulfate were obtained from Cambridge isotope laboratories (www.isotope.com). FPLC Sephacryl S-200 was obtained from Pharmacia LKB (Piscataway, NJ).

### Methods—bacterial isolates and growth conditions

Industrial water samples from different geological areas of districts Kasur and Sheikhupura, Punjab, Pakistan were collected in sterilized polythene bags. All samples were brought to laboratory and different physico-chemical parameters were analyzed within 24–48 h. Bacterial load of the samples was determined by making serial dilutions of 10^−1^ to 10^−4^ from 1% initial water sample. In order to isolate the individual colonies, 100 μl of the 10^−3^ and 100^−4^ dilutions were spread on LB agar plates containing different concentrations of HgCl_2_ ranging from 1 to 20 μg/ml. The plates were incubated at 37°C for 24 h. The single isolated and HgCl_2_ resistant colonies were selected and re-streaked on new LB agar plates without HgCl_2_ to get purified colonies. All Hg-resistant and purified bacterial cultures were stored as glycerol stocks (30%) at−80°C.

### Biochemical and molecular characterization

Highly Hg-resistant isolate AZ-15 was biochemically characterized by different tests *viz*., Gram staining, shape, motility, spore formation, catalase, oxidase, oxygen requirement, MacConkey agar growth, indole production, methyl red, Voges-Proskauer, citrate (Simmon) agar growth, and H_2_S production by following the protocols of Cappuccino and Sherman (2008). The bacterial genomic DNA of AZ-15 was extracted by genomic DNA purification kit. The universal primers were used for the amplification of 16S rRNA gene using BIOER XP-thermal cycler (Normand, 1995). The amplification conditions for both genes were set as: 5 min at 95°C, 35 cycles of 1 min at 95°C, 1 min at 55°C, and 2 min at 72°C, with a final 5 min chain elongation at 72°C. The sequence results obtained from Macrogen sequencing core facility, Korea were checked through nucleotide blast and submitted to NCBI GenBank.

### Cloning of *mer*F gene into pET31b(+) vector

The *mer*F gene (wild type) of highly mercury resistant *Enterobacter* sp. AZ-15 was amplified using the primers given in Table 1. The amplified product of 246 bp was digested with the restriction enzymes *Xho*l and *Sph*l, and purified by gel extraction kit. The digested *mer*F gene was ligated with the *Xho*l-*Sph*l-cleaved expression vector pET31b(+) (Kuliopulos et al., 1987). The genetic codons encoding cysteine residues in the native sequence of *mer*F gene were mutated into codons encoding serine residues using site directed mutagenesis kit by designing two sets of primers (Table 1). Both sequences of *mer*F gene (*mer*F wild type and mutated *mer*F) along with translated peptide sequences were submitted to the NCBI database (www.ncbi.nlm.nih.gov/) and obtained accession numbers as MF185183 and MF185184, respectively. The designed recombinant plasmid containing mutated *mer*F (*mer*Fm) gene was transformed into DH5α competent cells and identified by using same restriction enzymes on agarose gel. The nucleotide sequence of recombinant pET31b(+) containing *mer*Fm gene was confirmed by DNA sequencing facility provided marketplace, University of California, San Diego (USA). Then the recombinant plasmid pET31b(+) was transformed into C43(DE3) competent *E. coli* cells. After transformation, stable C43(DE3): pET31b+*mer*Fm clones were screened and stored as glycerol stocks (20% of glycerol) at −80°C.

**Table 1:** Oligonucleotides for the amplification of 16S rRNA, wild type *mer*F and mutated *mer*F (*mer*Fm) genes.

**Gene**	**Primer pair**	**Primer sequence**
16S rRNA	16S-F	5′AGAGTTTGATCCTGGCTCAG3′
	16S-R	5′AAGGAGGTGATCCAGCCGCA3′
*mer*F	merF-F	5′ATCTAT**GCATGC**ATGAAAGACCCGAAGACACTGCTGCGGGTCAGC3′
	merF-R	5′ATATAT**CTCGAG** TCATTTTTTTACTCCATTGAATTTCGGGG3′
*mer*Fm	merFm-1	5′GTGGCGCTCAGTTCGTTCACCCCTGTTCTGG3′
	merFm-1	5′CCAGAACAGGGGTGAACGAACTGAGCGCCAC3′
	merFm-2	5′CAAGCCGATGCCTCGTCCACCCCGAAATTCAAT3′
	merFm-2	5′ATTGAATTTCGGGGTGGACGAGGCATCGGCTTG3′

*Bold sequences are restriction sites of specific enzymes*.

### Production of ^15^N-labeled recombinant MerFm

The expression of recombinant *mer*Fm gene was optimized in LB medium and unlabeled M9 medium supplemented with different concentrations (0.2, 0.5, and 1 mM) of isopropyl-β-D-thiogalactoside (IPTG) at different time intervals (2, 4, 6, 7 h, and overnight) of incubation. For expression of isotopically labeled MerFm protein, 5 ml of LB medium with final concentration of 50 mg/l of cabenicillin was inoculated with 5 μl of clone C43(DE3): pET31b+*mer*Fm glycerol stock. After 5 h incubation at 37°C, 1 ml of starter culture was used to inoculate 50 ml of M9 minimal medium (per liter) containing Na_2_HPO_4_ 7.0 g, KH_2_PO_4_3.0 g, NaCl 0.5g, CaCl_2_ 0.1 mM, MgSO_4_ 1 mM, thiamin 50 mg, d-glucose 10 g, and ^15^N-(NH_4_)_2_SO_4_ 1 g containing 50 mg/l of carbenicillin. The culture was kept at 300 rpm at 37°C for overnight and 50 ml culture was poured into 450 ml of same ^15^N labeled-M9 medium. The cells were allowed to grow at 37°C in shaking incubator until the optical density (O.D_600_) of 0.6 was achieved. The expression of recombinant plasmid (KSI_MerFm_His-tag) was achieved by adding 1 mM of IPTG as a final concentration and kept on shaking for another 7 h on same conditions. The pellet of C43(DE3) cells containing recombinant protein was obtained by subsequent harvesting at 7,000 rpm for 30 min at 4°C.

### Purification of the recombinant protein by Ni-NTA chromatography

The cell pellets were resuspended in 30 ml lysis buffer (50 mM Tris hydrochloride, 15% glycerol (v/v), 1 mM NaN_3_, pH 8.0). After 10 min of incubation at room temperature, cell lysate was sonicated by a probe sonicator (www.fishersci.com) for 5 min (cycles: 5 s on/10 s off) on ice and then centrifuged at 17,000 rpm for 30 min at 4°C. The pellet at this stage contains purified inclusion bodies containing more than 90% of MerFm fusion protein. The pellet containing inclusion bodies was then resuspended in the nickel column binding buffer (20 mM Tris hydrochloride, 500 mM NaCl, 6 M guanidinium hydrochloride (GndCl), 5 mM imidazole, pH 8.0) by tip sonication, and centrifuged again at 19,000 rpm for 1 h to remove remaining protein, lipids, and lipid-associated debris. The supernatant was loaded onto a Ni-NTA His-Bind Resin column, pre-equilibrated with binding buffer. The column was then washed with washing buffer (20 mM Tris hydrochloride, 500 mM NaCl, 6 M guanidinium hydrochloride (GndCl), 50 mM imidazole, pH 8.0). The fusion protein was eluted with elution buffer (20 mM Tris hydrochloride, 500 mM NaCl, 6 M GndCl, 500 mM imidazole, pH 8.0) and concentrated to 30 ml using an Amicon stirred concentrator cell with YM10 filter membrane and dialyzed against ddH_2_O in a 10,000 kDa MWCO dialysis membrane with four water changes until the protein precipitates out of solution. The precipitated protein was then lyophilized.

### Cyanogen bromide cleavage of the fusion protein

The lyophilized protein was dissolved in 70% formic acid solution and cleaved by addition of a three-fold excess of cyanogen bromide (CNBr) for 3–5 h in the absence of light. The cleavage reaction was stopped by adding double volume of 1M NaOH. The cleaved polypeptide was dialyzed by using 3,500 kDa MWCO dialysis membrane against water and then lyophilized.

### Purification of MerFm by FPLC

Cleaved protein was re-dissolved in 4 ml phosphate SDS buffer (100 mM Na_2_HPO_4_, 4 mM SDS, I mM EDTA, lmM NaN_3_, pH 8.2) and 1 ml 1 N NaOH. Size-Exclusion gel chromatography using a Sephacryl S-200 column equilibrated with phosphate-SDS buffer on a Pharmacia FPLC system used to separate the KSI fusion partner from the pure MerFm protein. Fractions containing MerFm were pooled and concentrated down to 30 ml using an Amicon Stirred cell with a 3,500 MWCO membrane. The pure protein was then dialyzed against ddH_2_O (with 40 mM β-mercaptoethanol for native MerFm) to remove SDS for six times with time interval of 12 h until the protein precipitates. Each time the protein was centrifuged out of solution, quickly lyophilized, and stored at −20°C for further use, Yields of MerFm were approximately 2–3 mg/l of cell culture. SDS-PAGE was run to monitor the purification, with the final native MerFm protein.

### NMR spectroscopy of MerFm

All NMR samples were prepared by resolubilizing the lyophilized protein in NMR buffer (10 mM Na_2_HPO_4_, 500 mM d_25_-SDS, lmM NaN3, pH 6.0). The protein was then incubated at 40°C for 30 min with intermittent bath sonication and centrifuged at 13,000 rpm for I5 min to remove undissolved protein and other debris. The labeled peptide samples were prepared in different concentrations 10, 40, and 75% of D_2_O. Two-dimensional heteronuclear single quantum coherence (HSQC) spectra of uniformly ^15^N-labeled MerFm were recorded on Bruker DMX 600 spectrometer equipped with a triple resonance probe, three-axis pulsed field gradients, a deuterium lock channel and operated at a ^1^H NMR frequency of 600 MHz with total recording time 7 h. The other parameters for obtaining ^15^N–^1^H fast Heteronuclear Single Quantum Cohererance (fHSQC) spectra include pulse sequence with 1,024 points in t2 and 256 points in t1 and 50°C using a 1.5 s recycle delay. The spectra were processed by using Sparky 3 [Goddard and Kneller, University of California San Francisco (UCSF), USA].

### Detoxification of Hg^2+^ by bacteria and SEM analysis

An experiment was designed to check the detoxification potential of *Enterobacter* sp. at a lab scale. Mercury resistant (Hg^R^) bacterial strain AZ-15 and mercury sensitive strain (Hg^S^) *Enterobacter cloacae* ZA-15 (KJ728671) with equal number of cells (cfu/ml) were inoculated separately in flasks containing 30 mL of LB medium, supplemented each flask with 20 μl/ml of HgCl_2_ to determine the detoxification efficiency of mercury. The flasks were incubated at 37°C for up to 8 h with 2 h of time intervals at 120 rpm of agitation in triplicates. After incubation, cultures were spun down at 14,000 rpm for 15 min and detoxification of Hg^2+^ was estimated by dithizone method (Elly, 1973; Khan et al., 2006). For scanning electron microscopy (SEM), one drop of 48 h culture was diluted into 15 ml of ddH_2_O and dried on carbon surface slide. The topographical, morphological and crystallographic information of bacterial cell membrane was monitored using Scanning Electron Microscope (SEM) (JEOL-JSM-6480).

### Statistical analysis

The phylogenetic analysis of *Enterobacter* sp. AZ-15 with already reported bacteria on the basis of 16S rRNA gene was done by Mega 6.0 software (Tamura et al., 2013). The results of lab scale mercury detoxification experiment were subjected to mean, standard deviation, analysis of variance (ANOVA) by using SPSS V.20 software (IBM, 2011). The hydropathy plot predicting membrane spanning regions was built by SWISS-MODEL homology (www.expasy.org).

## Results

### Screening of Hg-resistant bacteria

Industrial water samples collected from different geological sites (tanneries) of district Kasur and Itehad chemicals limited, district Sheikhupura, Lahore were checked for physico-chemical properties such as temperature, pH, Hg^2+^ concentration, bacterial load in the presence of metal stress of HgCl_2_. Data indicate the level of pH in water sample falls in the range of 6.47–7.65. All samples (100%) were within the pH ranges of WHO drinking water guidelines (WHO, 2004) but the value of HgCl_2_ which range from 5to 17 μg/ml has crossed limit described by WHO (1 μg/ml) (Javendra, 1995). The bacterial count in these samples is also variable, ranging from 2.1 × 10^2^ to 2.9 × 10^5^ as presented in Table 2. A total 30 bacterial strains including Gram positive and Gram negative were screened on the basis of showing resistance against different concentrations of HgCl_2_.

**Table 2:** Level of physico-chemical parameters and bacterial load in industrial water samples.

**Samples**	**Temperature**	**pH**	**Bacterial load**	**Hg^2+^ (μg/ml)**
Officers colony	28.5	6.47	3.02 × 10^3^	1
Gulberg Town	30.5	7.45	2.90 × 10^5^	5
Civil Hospital	29	7.23	3.00 × 10^4^	8
Nafees colony	33.5	7.65	2.90 × 10^4^	1
Itehad chemicals	34	7.25	2.1 × 10^2^	2
**WHO guidelines for drinking water quality**				
	………	6.5–8.5	………	1

### Biochemical and 16S rDNA identification

The most highly Hg-resistant bacterial isolate AZ-15 showing growth at 20 μg/ml of HgCl_2_, was characterized on the basis of colony morphology and biochemical tests. The results are shown in Table 3. Mercury resistant bacterial isolate was identified by 16S rDNA sequencing (≈1.5 kb) as *Enterobacter* sp. with accession number KU558920. Other close matches to *Enterobacter* sp. AZ-15 (KU558920) includes *E. cloacae* strain E717 (EF059865), *Enterobacter* sp. BSRA3 (FJ868807), *E. cloacae* strain NBRC 13535 (NR113615), *E. cloacae* strain DSM 30054 (NR117679), *E. cloacae* strain RPR-CCFL3 (KR611993) and *Enterobacter* sp. Wy2-D9 (JN986806). *Enterobacter* sp. GJ1-11 (EU139848), *Enterobacter* sp. BAB-3140 (KF984440), and *Enterobacter* sp. STJ12 (KC833508) represent as an out group in this phylogenetic analysis (Supplementary Figure 1).

**Table 3:** Results of biochemical tests of bacterial isolate AZ-15.

**Biochemical tests**	**Results**
Gram staining	Gram-ve
Shape	Rods
Motility	Motile
Spore formation	Non-spore forming
Catalase	Positive
Oxidase	Negative
Oxygen requirement	Facultative anaerobe
MacConkey growth	Positive
Indole production	Negative
Methyl red	Negative
Voges-Proskauer	Positive
Citrate (Simmon)	Positive
H_2_S production	Negative
AZ-15	*Enterobacter* sp.

### Plasmid construction

**Figure 1:**
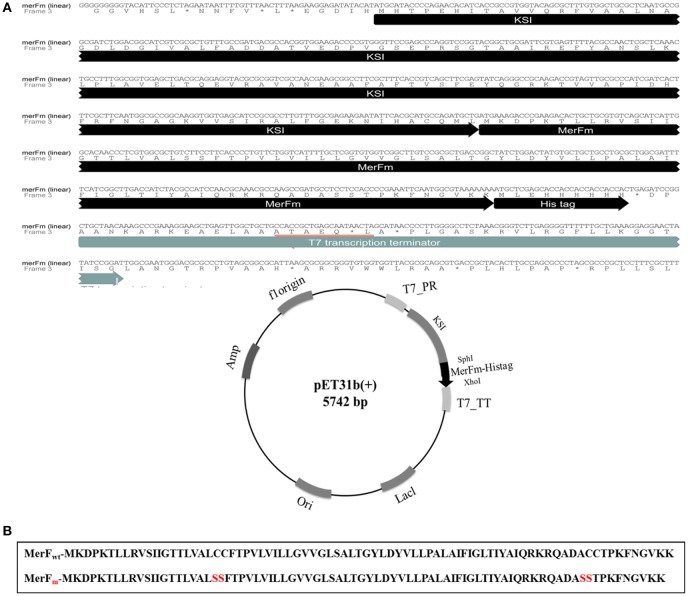
**(A)** Linear and circular forms of nucleotide and amino acid sequences of pET31b+ plasmid with *mer*Fm gene. T7_PR, T7 promoter; T7_TT, T7 terminator; His-tag_KSI, fusion partner; *Sph*I and *Xho*I, restriction enzyme sites; ori, replication **(B)** Amide sequences of MerF protein before and after site-directed mutagenesis.

As shown in Figure 1A, the gene construct *mer*Fm was designed by ligation of wild type *mer*F gene in pET31b(+) vector. The deduced amino acid sequence of *mer*F gene showed two cysteine residues at the positions 71 and 72. The cysteine residues were mutated into serine residues in *mer*Fm gene by site-directed mutagenesis which results no cysteine residue in the final *mer*Fm gene construct as shown in Figure 1B. The salient features of construct include T7 promoter, ketosteroid isomerase (KSI) fusion partner, 5′ overhanging sequences compatible with *Sph*I and *Xho*I restriction enzyme sites, within restriction sites; a transcription initiation codon (ATG) of *mer*Fm followed by encoding region of respective protein and a termination codon and a region encoding a hexahistidine amino acid tag and T7 terminator. His-tag sequence is for ease of purification and the KSI peptide sequence is designed to form inclusion bodies. After the transformation of recombinant plasmid into competent DH5α *E. coli* cells, the plasmid DNA was purified at concentration of 45 mg/ml. The plasmid was then transformed into *E. coli* C43(DE3) cells for expressing high concentration of MerFm recombinant protein and is thus protected from proteolysis. The fusion protein is non-toxic to the *E. coli* host cell, and is expressed at levels up to 20% of total cellular protein in *E. coli* strain C43(DE3). This approach to the production of MerFm in *E. coli* may be generally applicable to other membrane proteins. We have used it successfully with several other small membrane proteins.

### Expression and purification of ^15^N-labeled MerFm protein

**Figure 2:**
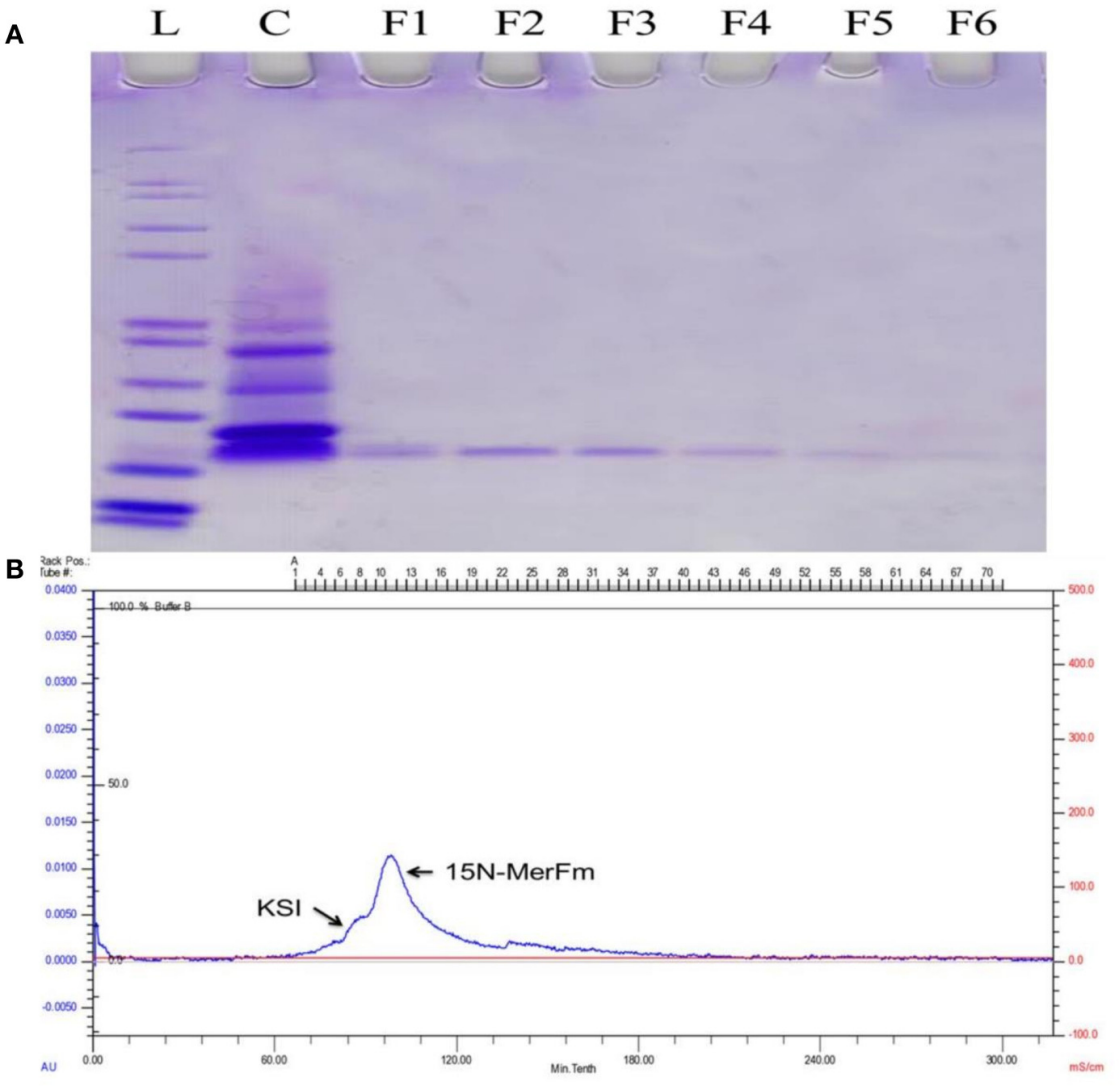
**(A)** L: Molecular weight (kDa) ladder. C: cleavage of fusion protein. F1–F5: Purified fractions of MerFm from FPLC column. **(B)** FPLC chromatogram showing peaks of native MerFm and fusion partner KSI.

For optimization of recombinant protein MerFm, three different concentrations, 0.2, 0.5, and 1 mM of IPTG were used at different post-induction time (h) viz., 2, 4, 6, 7, and overnight. The high expression of recombinant protein MerFm was achieved with 1 mM of IPTG and 7 h of post-induction time as shown in lane 12 of Supplementary Figure 2. The same conditions were maintained for expression of ^15^N-labeled MerFm fusion protein. After expression, the separation and purification of ^15^N-MerFm were performed by following the procedure described in detail in the methodology section. Briefly, the recombinant/fusion protein formed as inclusion bodies was separated from the total cell lysate by centrifugation. The fusion protein was partially purified by nickel affinity chromatography. The data in Supplementary Figure 3, illustrate the expression and isolation of inclusion bodies of full-length MerFm by Ni-NTA column. Lane 1 is showing Mark12™ unstained protein ladder (www.lamdabio.com). After the post-induction period of 7 h, the cells were lysed by sonication and centrifuged. After centrifugation, the supernatant was discarded which contained the total cellular proteins except inclusion bodies is shown in lane 2 of a 12% SDS-PAGE. Lane 3 contains the inclusion bodies solubilized in binding buffer and lanes 4 and 5 show the soluble fraction (flow through) and washing of other proteins with washing buffer, respectively except the targeted fusion protein. The lanes 6–10 show that the insoluble fractions (inclusion bodies) containing primarily the targeted fusion protein. After elution from the nickel column, the KSI fusion partner was cleaved from the MerFm polypeptide in the presence of CNBr at methionine for obtaining purified native MerFm. The lane 2 in Figure 2A shows the cleavage of fusion protein and the lane 3 contains the little bit impurity of KSI fusion partner while lanes 4–8 show the purified fractions ^15^N-MerFm after size-exclusion chromatography (FPLC). Figure 2B shows the peaks of ^15^N-MerFm and KSI in FPLC.

### NMR spectra of ^15^N-MerFm

**Figure 3:**
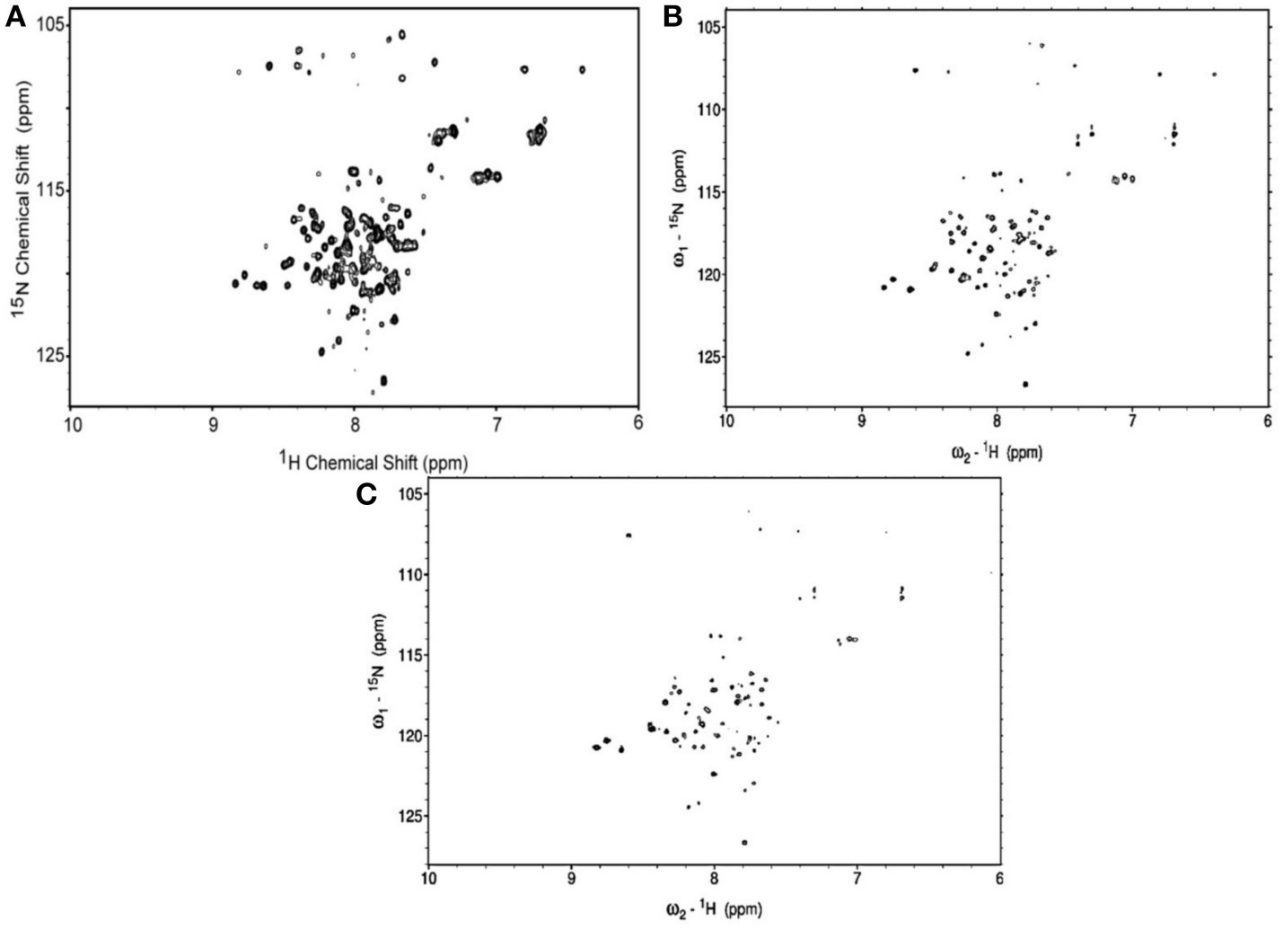
^1^H–^15^N HSQC spectra of uniformly-15N-labeled MerFm in SDS micelles with different concentrations of D_2_O **(A)** 10% **(B)** 40%, and **(C)** 75%.

The ^1^H-^15^N HSQC spectra of MerFm were obtained with the three concentrations of D_2_O as shown in Figure 3. The high quality spectrum of 15N-MerfM was obtained with 40% concentration of D_2_0 (Figure 3B). 1H-15N HSQC spectra were recorded under standard solution NMR conditions to confirm the purity and correct folding of the MerFm protein. The two-dimensional HSQC spectrum of ^15^N enriched MerFm contains the correct number of crosspeaks/resonances for each amide in the protein corresponding to side chain and tryptophan amide groups. This confirms that no modification of the tryptophan residue occurred during CNBr cleavage. Notably, the crosspeaks of six glycines in full-length MerFm are also present in the spectrum (Figure 3B). This is prerequisite for the preparation of well-behaved protein in phospholipid bilayer samples for solid-state NMR studies.

### Detoxification of Hg^2+^ and SEM analysis

**Figure 4:**
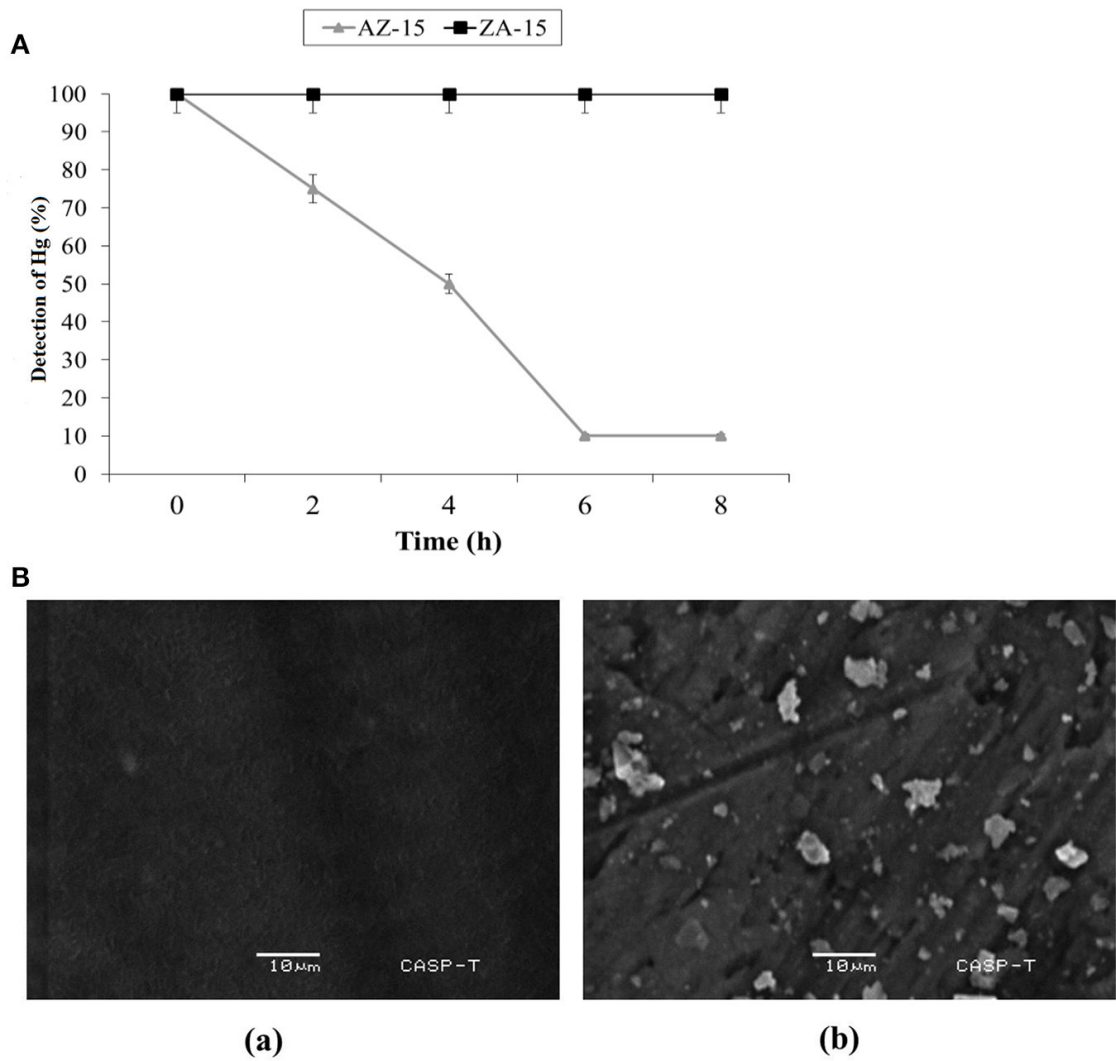
**(A)** Detoxification of Hg in LB medium. X-axis shows the given incubation time (h) and Y-axis shows the % of Hg detected at different time intervals **Ba**, SEM analysis of Hg-resistant *Enterobacter* sp. AZ-15 **Bb**, Hg-sensitive *Enterobacter cloacae* ZA-15.

In lab scale experiment, the detoxification of Hg^2+^ by selected bacterial strain indicated 5 (25%), 10 (50%), 18 (90%), and 18 (90%) μg/ml detoxification of Hg^2+^ out of 20 μg/ml (100%) at 2, 4, 6 and 8 h of incubation, respectively by mercury resistant *Enterobacter* sp. AZ-15 (*p* < 0.05). The mercury sensitive strain *E. cloacae* ZA-15 was used as a negative control (Figure 4A). In SEM analysis, the accumulation of Hg was seen on the cell surface after 8 h of incubation with 20 μg/ml in LB medium. The SEM image showed crystalline structures of Hg deposited on the surface of cell membrane while in negative control, no crystalline structure was observed (Figure 4B). From this experiment, it is evident that *Enterobacter* sp. AZ-15 can accumulate Hg which confirms its detoxification potential.

## Discussion

Mercury is widespread in our environment. This is not only a result of anthropogenic activities but also due to various geochemical and biological processes. Hence, nature provided a rich toolbox for transformation of mercury. It includes the microbial mercury resistance operon, which carries genes for binding, transport, and detoxification of Hg^2+^ and organomercurials. Among these tools, the transportation of mercury through microbial membranes plays a crucial role in the bacterial mercury detoxification system (Barkay et al., 2003; Parks et al., 2013).

Four families of integral membrane proteins (IMPs) have been identified in the characterized *mer* operons, ranging from 2 to 4 predicted transmembrane elements: MerE, MerF, MerT, and MerC. Across the mer operons, MerT, a tri-spanning, 116 residue protein, is considered to be the principal mercury transporter and has been shown to be an essential component in most mer operons capable of bestowing mercuric resistance. The extent to which the other IMPs, MerF and MerE, are capable of functioning independently of MerT has not yet been conclusively determined (Mok et al., 2012).

In previous studies, the full length and truncated structures of MerF have been deeply studied by NMR spectroscopy found in *E. coli* and in plasmid pMER327/419 of *Pseudomonas fluorescens* between *mer*P and *mer*A (Veglia and Opella, 2000; Wilson et al., 2000). This is the first study to analyze the gene construction, expression and purification of mutated MerF (MerFm) amplified from *Enterobacter* sp. AZ-15. The bacterium, *Enterobacter* sp. AZ-15 isolated from mercury contaminated industrial effluent samples, could resist Hg^2+^ upto 20 μg/ml. In other reports, the potential of *Enterobacter* sp. to tolerate Hg^2+^ varied (Table 4). Phylogenetic analysis of this bacterium AZ-15 showed 98% similarity to already reported *Enterobacter* species (Supplementary Figure 1). A gene construct of mutated *mer*F was designed and expressed with 1 mM IPTG and 7 h post-induction at 37°C. The cysteine residues in wild type MerF sequence induce aggregation due to disulfide bonds which makes the protein difficult to purify.

**Table 4:** Mercury resistant bacteria and their patterns of resistance to different concentrations of Hg^2+^ in different media.

**Bacteria**	**MIC**	**Media used**	**References**
*Pseudomonas putida* SPi3	NR	LB	Von Canstein et al., 1999
*Psychrobacter* sp. ORHg1	100 μM (20.06 μg/ml)	NEM	Pepi et al., 2011
*Pseudomonas putida* SP1	300 μM (60 μg/ml)	2216E	Zhang et al., 2012
*Bacillus cereus*	30 μM (6 μg/ml)	LB	François et al., 2012
	15 μM (3 μg/ml)	PB	
*Lysinibacillus* sp.	60 μM (12 μg/ml)	LB	François et al., 2012
	15 μM (3 μg/ml)	PB	
*Kocuria rosa*	20 μM (4 μg/ml)	LB	François et al., 2012
	30 μM (6 μg/ml)	PB	
*Microbacterium oxydans*	100 μM (20 μg/ml)	LB	François et al., 2012
	30 μM (6 μg/ml)	PB	
*Serratia marcescens*	60 μM (12 μg/ml)	LB	François et al., 2012
	30 μM (6 μg/ml)		
*Ochrobactrum* sp.	60 μM (12 μg/ml)	LB	François et al., 2012
	30 μM (6 μg/ml)		
*Ensifer medicae*	6 μM (1.2 μg/ml)	YEM	Ruiz-Díez et al., 2012
*Rhizobium leguminosarum*	12.5 μM (2.5 μg/ml)	YEM	Ruiz-Díez et al., 2012
*Rhizobium radiobacter*	30 μM (6 μg/ml)	YEM	Ruiz-Díez et al., 2012
*Bradyrhizobium canariense*	12.5 μM (2.5 μg/ml)	YEM	Ruiz-Díez et al., 2012
*Providencia alcalifaciens*	9.2 μM (1.84 μg/ml)	LB	Cabral et al., 2013
*Pseudomonas putida*	11.5 μM (2.3 μg/ml)	LB	Cabral et al., 2013
*Enterobacter* sp. AZ-15	100 μM (20 μg/ml)	LB	This study
*Sphingobium* sp. SA2	25.43 μM (5.1 μg/ml)	LP	Mahbub et al., 2016
	220 μM (44.15 μg/ml)	NB	
*Enterobacter* sp. A25B	400 μM (80 μg/ml)	LB	Giovanella et al., 2016
*Enterobacter* sp. B50C	250 μM (50 μg/ml)		
*Bacillus* sp. AZ-1	100 μM (20 μg mL^−1^)	LB	Amin and Latif, 2016
*Bacillus cereus* AZ-2	100 μM (20 μg mL^−1^)		
*Enterobacter cloacae* AZ-3	50 μM (10 μg mL^−1^)		

Moreover, the MerFm was purified by fast protein liquid chromatography (FPLC) but Lu and Opella (2014) used high performance liquid chromatography (HPLC) for purification of wild type and truncated MerF. The high quality spectra of ^1^H-^15^N HSQC confirmed full expression of the native sequence of MerFm and each amino acid (with the exception of proline) contains one ^1^H-^15^N unit in the backbone. The hydropathy plot (Supplementary Figure 4) suggests that MerF has two hydrophobic trans-membrane helices, although the N-terminal portion of the plot is somewhat atypical. Wild-type MerF has two pairs of vicinal Cys residues (replaced by serines in MerFm), which is unusual, and contrasts with the -CXXC- sequences typically found in proteins that bind mercury and other heavy metals (Opella et al., 2002). The vicinal arrangements of the cysteines have profound consequences for their metal-binding properties and the selectivity for Hg^2+^ over other metals (DeSilva et al., 2002). With both metal transfer and transport activities, the structural biology of MerF is too elaborate to be explained simply by Hg^2+^ binding to pairs of cysteines. Therefore, it is essential to determine its three-dimensional structure in phospholipid bilayers, where it executes its functions. Experiments are underway to explore further structural properties of MerFm having natural origin.

The capability of different bacterial species to detoxify Hg^2+^ varies in different Hg supplemented enriched media as shown in Table 4. The present study also determined that the *mer*F gene in *Enterobacter* sp. AZ-15 encodes a polypeptide sequence capable of proper folding, NMR studies and also ensures the presence of *mer* operon. The favorable thermodynamic and spectral characteristics of the recombinant protein will allow the detailed description of this natural protein. During the investigation of Hg-detoxification capability, *Enterobacter* sp. AZ-15 showed 90% detoxification potential of Hg^2+^ from HgCl_2_ supplemented LB medium. The crystalline structures of deposited Hg were visualized by SEM. Sathyavathi et al. (2013) and Mahbub et al. (2016) have previously reported the SEM analysis of same crystalline structures of detoxified Hg.

In conclusion, the expression and purification of ^15^N-labeled MerFm from mercury resistant *Enterobacter* sp. AZ-15 (MIC-20 μg/ml), was first time successfully achieved. ^1^H–^15^N HSQC NMR spectra confirmed the expression of exact number of all residues of MerFm by producing one cross peak for each ^1^H–^15^N pair in a protein. In lab scale experiment, *Enterobacter* sp. AZ-15 showed 90% bioremediation of Hg from Hg-supplemented LB medium. On the basis of these characteristics, mercury resistant *Enterobacter* sp. AZ-15 could be used as a potential candidate to deteriorate the toxic effects of Hg^2+^ from the environment.

## Author contributions

AA conducted the experiments and wrote the paper while ZL proofread and overall supervised the project.

### Conflict of interest statement

The authors declare that the research was conducted in the absence of any commercial or financial relationships that could be construed as a potential conflict of interest.
