# Supplementary material for: Cloning, Expression, Isotope Labeling, and Purification of Transmembrane Protein MerF from Mercury Resistant Enterobacter sp. AZ-15 for NMR Studies
Source: Front Microbiol. 2017 Jul 7;8:1250. doi: 10.3389/fmicb.2017.01250 (PMC5500634; doi:10.3389/fmicb.2017.01250)
Supplement: Supplementary file 1 [file DataSheet1.docx]

Supplementary Material

**Cloning, Expression, Isotope Labelling, and Purification of Transmembrane Protein MerF from Mercury Resistant *Enterobacter* sp. AZ-15 for NMR Studies**

Aatif Amin* and Zakia Latif

Department of Microbiology and Molecular Genetics, University of the Punjab, Lahore-54590, Pakistan

***For correspondence: **Aatif Amin,** E.mail: [aatifamin93@gmail.com](mailto:aatifamin93@gmail.com)

## Supplementary Figure

##
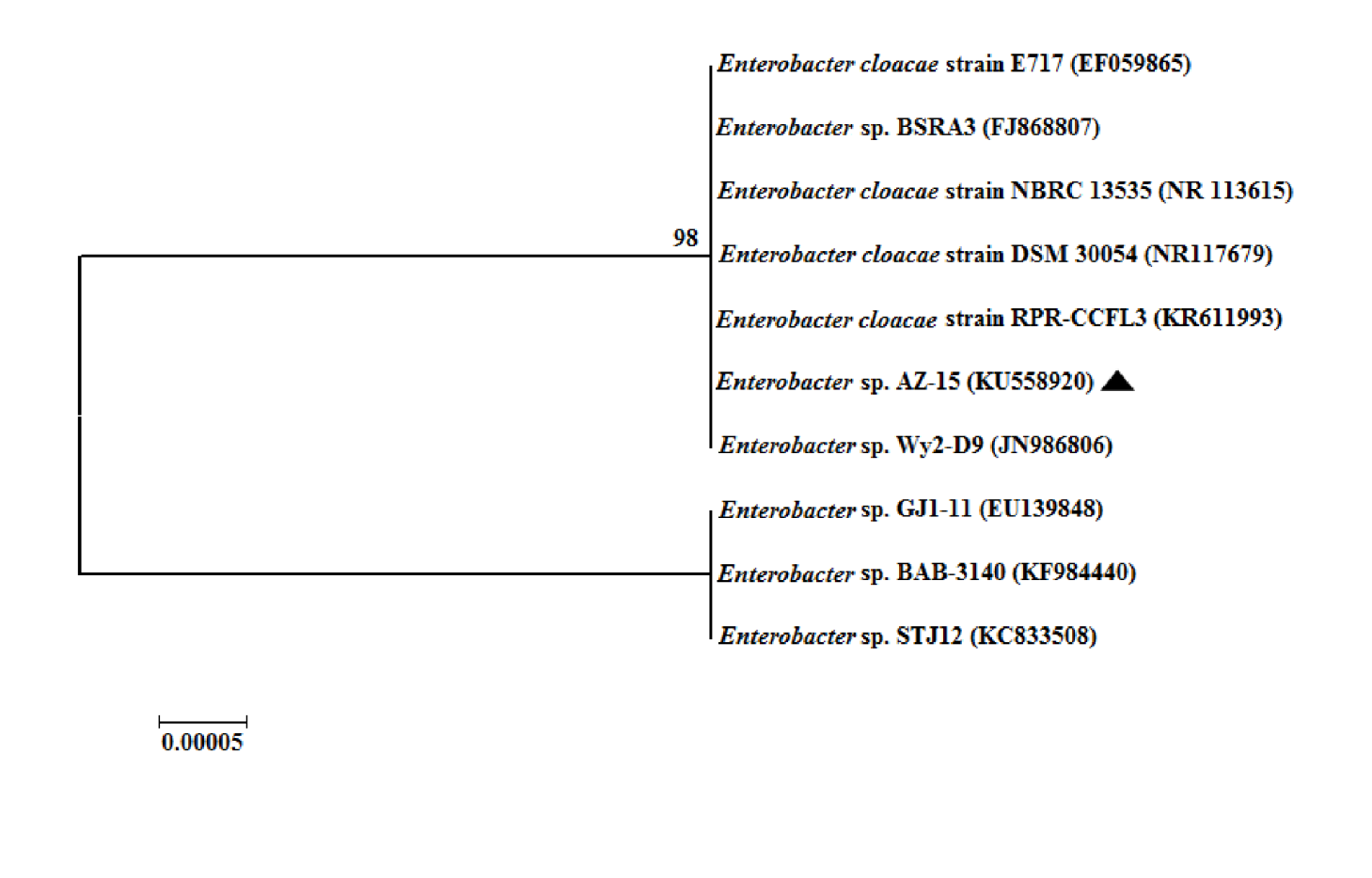


## Supplementary Figure 1. Phylogenetic relationship of *Enterobacter* AZ-15.The multiple sequence alignment through clustalW was used to check phylogeny among bacterial species of *Enterobacter* genera on the basis of 16S rRNA gene through neighbor joining method using MEGA 5 software. The percentage of homology among different clade was checked with bootstrap test at value of 1000 replica as shown next to the branches. The tree is drawn to scale which represent the nucleotide change. The units of branch lengths and the evolutionary distances are same, used to infer the phylogenetic tree.


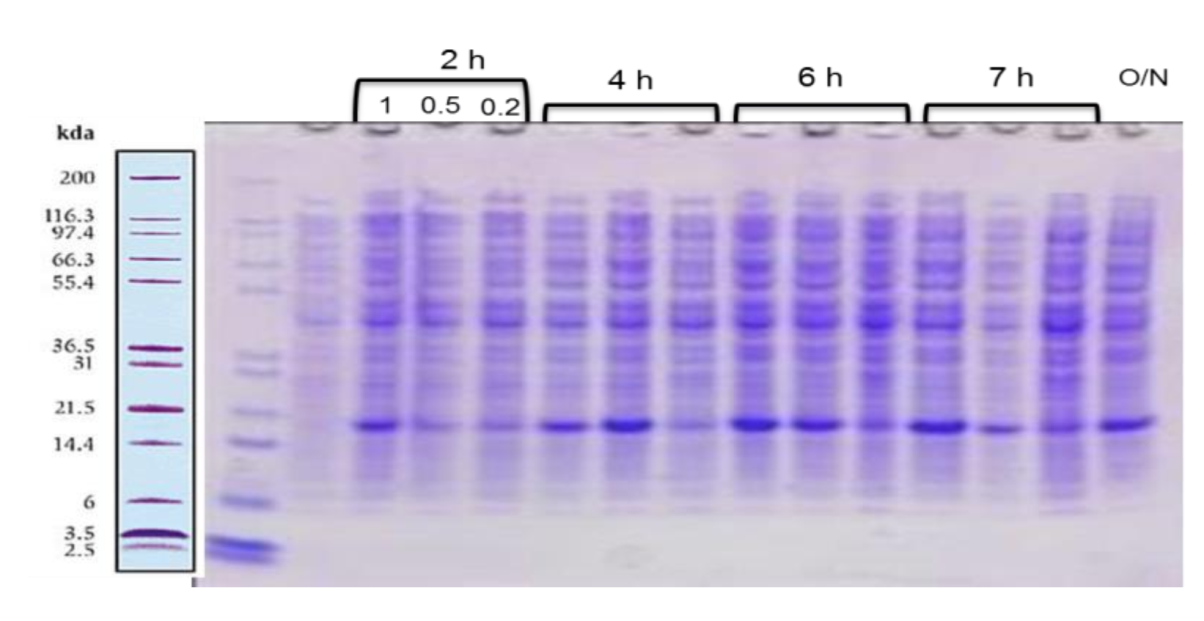


**Supplementary Figure 2.** Optimization of recombinant MerFm protein expression at different concentrations of IPTG (0.2, 0.5 and 1mM ) and at different post-induction time (2, 4, 6, 7 h and overnight).

**
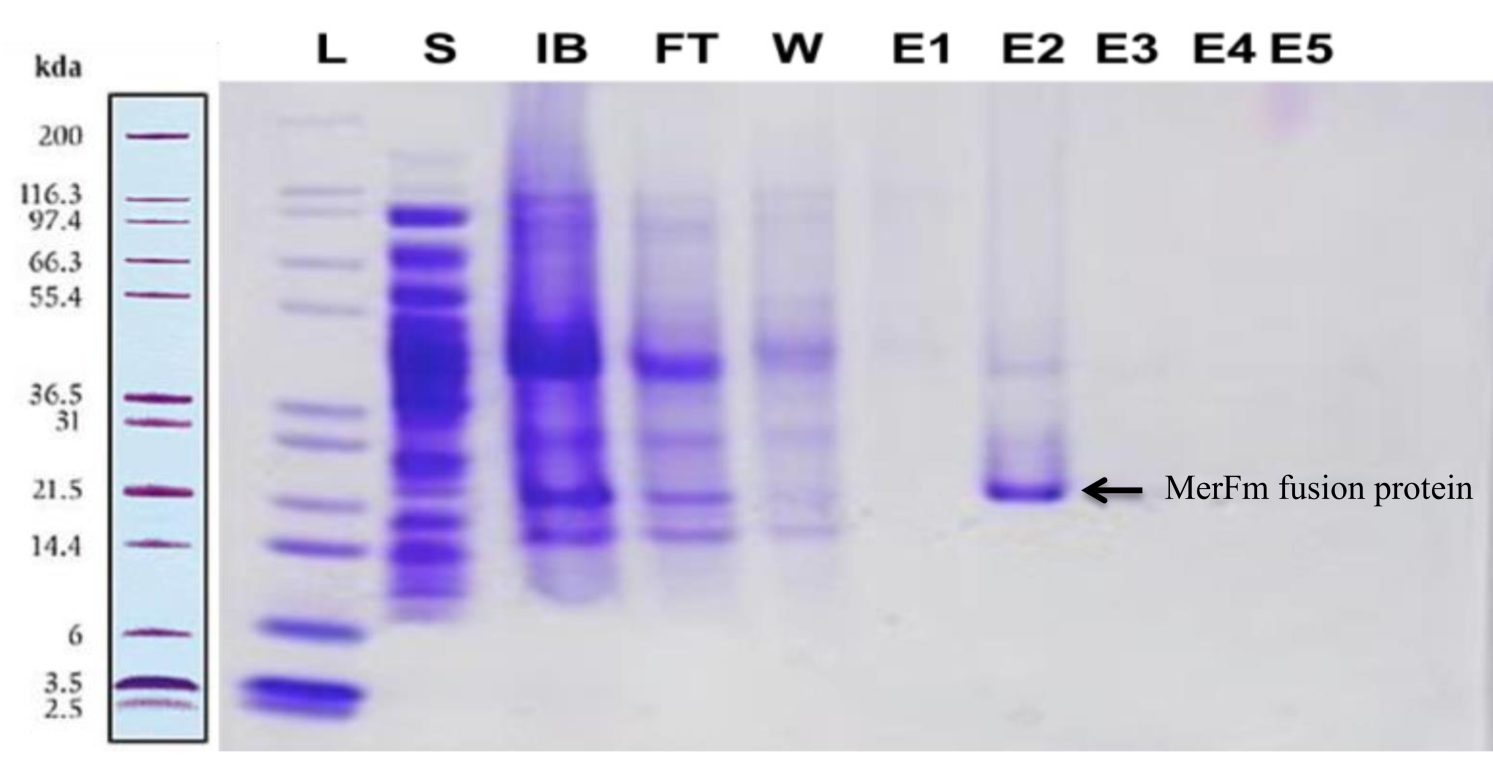
**

**Supplementary Figure 3.** SDS–PAGE of the expressed MerF_m_ fusion protein in *E. coli* strain C43(DE3). L: Molecular weight ladder. S: supernatant. IB: Inclusion bodies. FT: Flow through. W: Washing. E1-E5: Elution fractions from Ni-NTA column.

**
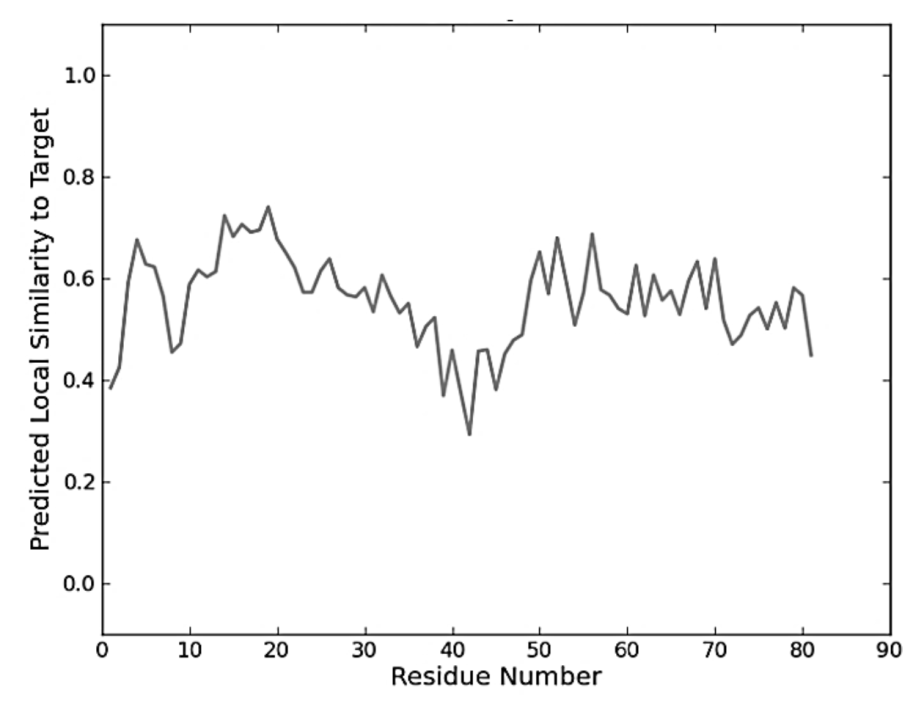
**

**Supplementary Figure 4.** The hydropathy plot predicting the numbers of membrane spanning regions in protein MerFm ([Howell et al., 2005](#_ENREF_1)).

**References**

Howell, S.C., Mesleh, M.F., and Opella, S.J. (2005). NMR structure determination of a membrane protein with two transmembrane helices in micelles: MerF of the bacterial mercury detoxification system. *Biochemistry* 44(13)**,** 5196-5206.
